# A rapid and sensitive assay to identify HLA-DQ2/8 risk alleles for celiac disease using real-time PCR method 

**Published:** 2018

**Authors:** Kazem Mashayekhi, Mohammad Rostami-Nejad, Davar Amani, Mostafa Rezaei-Tavirani, Hamid Mohaghegh-Shalmani, Mohammad Reza Zali

**Affiliations:** 1 *Department of Celiac Disease, Gastroenterology and Liver Diseases Research Center, Research Institute for Gastroenterology and Liver Diseases, Shahid Beheshti University of Medical Sciences, Tehran, Iran.*; 2 *Immuno-Biochemistry lab, Immunology Research Center, Mashhad University of medical Sciences, Mashhad, Iran*; 3 *Department of Immunology, Medical school, Shahid Beheshti University of Medical Sciences, Tehran, Iran*; 4 *Proteomics Research Center, Faculty of Paramedical Sciences, Shahid Beheshti University of Medical Sciences, Tehran, Iran *

**Keywords:** Celiac disease, Real-time PCR, Melting curve analysis, HLA-DQ2/8 alleles, HLA typing

## Abstract

**Aim:**

To perform a simple, rapid and sensitive Real-time PCR based SYBR Green method to determine the human leukocyte antigen (HLA)-DQ 2/8 alleles in celiac disease (CD) patients.

**Background:**

Many molecular techniques are available to determine the HLA-DQ2 and DQ8 alleles, but they are too expensive and have many steps that make them difficult to use.

**Methods:**

To determine the HLA-DQ 2/8 alleles we have developed a new real-time PCR assay, using SYBR Green technique with melting curve analysis on genomic DNA isolated from 75 CD patients and 94 healthy controls. The specific primers to examine HLA-DQA1*05, HLA-DQB1*02 and HLA-DQB1*0302 alleles were used and results were compared with commercially available kits.

**Results:**

Using this method, the presence of HLA-DQ2 and HLA-DQ8 alleles were determined with sensitivity and specificity 80% and 100% respectively and compared to low resolution commercially available kits, the results of this method were more efficient. The frequency of DQ2 and DQ8 in patients was 76% and 29%, respectively and overall 96% of patients were carries DQ2 and/or DQ8 alleles.

**Conclusion:**

The result of this study showed that Real-time PCR using SYBR Green method with melting curve analysis has good efficiency to identify the HLA-DQ2/8 risk alleles.

## Introduction

 Celiac disease (CD) is a polygenic and multifactorial disorder that is characterized by malabsorption of nutrients in small intestine and triggered by gluten a protein of wheat or related proteins from rye and barley. Symptoms varies from gastrointestinal to extra intestinal symptoms (1, 2). This disorder is very common with a mean prevalence of 0.5%–1% in the general population of Europe, United State as well as Iranian population (about 1%) (3-6). Both genetic and environmental factors are the main cause of CD development. Ingestion of gluten and human leukocyte antigen (HLA) are the main known environmental and genetic factors involved in complexity of disease respectively (1, 7, 8). CD has strong association with HLA-DQ2 (DQA1*05/DQB1*02 alleles) and HLA-DQ8 haplotypes (DQA1*03/DQB1*0302 alleles) and the current literature suggest that most of the patient with CD carry HLA-DQ2 and/or HLA-DQ8 haplotypes (90-95% and 5-10%, respectively) (2, 9).

Based on previous studies and recommendation of ESPGHN^ (^European Society for Pediatric Gastroenterology, Hepatology and Nutrition^)^, genetic examination of these haplotypes is recommended to rule out CD diagnosis in atypical cases (10-15). Many molecular techniques are available to determine the HLA-DQ2 and DQ8 alleles, but these methods in addition to high cost, have many steps that make them difficult to use, therefore the aim of this study was to set up a simple, rapid and sensitive Real-time PCR based SYBR Green method to determine the HLA-DQ haplotypes in patients with CD determining the allele frequency of these haplotypes in Iranian population. 

## Methods

**Sample population**

Seventy-five confirmed CD patients (27 males and 48 females, median age 31.7 years, range 7–67 years) and Ninety-four controls (33 males and 61 females, median age 32.3 years, range 10–70 years) were enrolled in this study. Patients with CD had positive tTG (tissue Trans-Glutaminase) and/or EMA (Endo-Mysial Antibodies) antibodies and histology consistent with CD according to the Marsh classification (Marsh I-III) (16). Using a valid questionnaire, demographic data including age, sex, Gastrointestinal (GI) and non-GI symptoms, history of smoking, family history of CD and history of the other diseases were collected. The study was approved by the ethics committee of Gastroenterology and Liver Diseases Research Institute, Shahid Beheshti University of Medical Sciences and all participants signed a consent form.

**Primer design**

Primers for Real-time PCR amplification were designed from exon 2 of HLA-DQ2 and HLA-DQ8 haplotypes using sequence in International Immunogenetics project Database (https://www.ebi.ac.uk/ipd/imgt/hla/). In addition to HLA-DQ2 and HLA-DQ8 haplotype primers, we designed a pair primer for detection of HLA-DQ2.5 as a HLA risk allele for CD (6, 7). For verification of amplification, an internal control of Beta2-Microgloboulin (B2M) from Primer Bank (https://pga.mgh.harvard.edu/primerbank/) was used. Primer properties are shown in Table 1.

**DNA extraction, conventional PCR and Real-time PCR set up**

DNA was extracted from whole blood collected using salting out method. Sample DNA concentrations were adjusted to 100 ng/μl after being quantified at 260 nm by the Nanodrop ND-1000 Spectrophotometer. For correct determination of primers’ melting temperature (Tm), temperature gradient was performed by conventional PCR, and the cycle steps are shows in Table 2. For Real-time PCR SYBR Green, three separate reactions per sample were performed for HLA-DQ2, HLA-DQ8 and HLA-DQ2.5. First, mix test separately was prepared for number of samples and then they were divided to 96-well plate (MicroAmp® Optical 96-Well Reaction Plate). Each mix was prepared as a multiplex reaction by B2M as an internal control. Known negative and positive samples that had been genotyped by commercial kits were used as controls for each of our tests. In addition, a negative DNA sample was run in each reaction as a control for detection of DNA contamination and then each plate was loaded onto the ABI7500 Instrument (Applied Biosystems® 7300 Real-Time PCR System). After Real-time PCR, melting curve was analyzed by using SDS program version 1.4.1 (Applied Biosystems®). The amounts of mixed per sample and cycle conditions are shown in table 3 and 4, respectively.

**Table 1:** **.** Primer Properties

Genotype	Primer name	Sequence	Amplicon Length (kb)
DQ2	DQB1*02	F: 5' GG ACA GAG GTG CGC CGT CTT R: 5' GC TTT CCT CCG CTC GAT CAG G	160
DQ8	DQB1*0302	F: 5' CGT GCG TCT TGT GCG GAC C R: 5' CTG TTC CAG GCG TAC TCG GCA	123
DQ2.5	DQA1*05	F: 5' CAC GTC GGT GCC TCT TAT GTA R: 5' GAC TCA AGT TAT TGT GTT TTA GG	205
Control	B2M	F: 5' TGC CTC GTT CAT TGA TGT TGT ATCT R: 5' TCTC TGC TCC CTA CCA CCT AGT	82

B2M: Beta2-Microgloboulin; F: Foeward Primer; R: Reverse Primer; kb: kilobase

**Table 2:** PCR cycles that used for Tm setup

Cycle	Step	Temperature	Time (s)	Cycle
1	Hold	95	600	1
40	Denaturation	95	40	40
	Annealing	60-70	35	
	Extension	72	40	
1	Hold	72	600	1

**Table 3.** The amount of mixed per sample

Materials used	Amount used (µl)
SYBR Green Master Mix	10
Forward Primer DQA/B1 (100 pmol/µl)	1
Reverse Primer DQA/B1 (100 pmol/µl)	1
Forward Primer B2M (100 pmol/µl)	1
Reverse Primer B2M (100 pmol/µl)	1
Deionized Water	5
Sample DNA (100 ng/µl)	1
Total	20

B2M: Beta2-Microgloboulin; DQA/B1: HLA-DQA1*05/ DQB1*0302 or DQB1*02.

**Table 4.** Real-time PCR cycle conditions

Cycle	Step	Temperature	Time (s)	Cycle
1	Hold	95	30	1
35	Denaturation	95	5	35
	Annealing	65	34	
	Extension	60	34	
1	Melt Curve Step	95 to 60 to 95		1

**Table 5:** Patient’s demographic data

Parameters		Total (%)
Yes	12 (16%)	Smoking
No	63 (84%)	
GI symptoms	53 (70%)	The cause of visits to the doctor
Non-GI symptoms	22 (30%)	
Yes	32 (42.6%)	History of disease
No	43 (57.4%)	
Yes	10 (13.4%)	Family history of CD
No	32 (42.6%)	
Unknown	33 (44%)	
Heartburn	52 (69.4%)	GI symptoms
Diarrhea	36 (48%)	
Nausea and Vomiting	30 (40%)	
Bloating	62 (82.7%)	
Weight Loss	51 (68%)	Non-GI symptoms
Anemia	43 (57.4%)	
Bone problems	31 (41.4%)	
Neurological problems	46 (61.4%)	
Infertility	4 (5.4%)	
Aphthous	37 (49.4%)	
Skin problems	18 (24%)	
Marsh I	7 (9.3%)	Histology
Marsh II	9 (12%)	
Marsh IIIa	18 (24%)	
Marsh IIIb	13 (17.3%)	
Marsh IIIc	28 (37.4%)	

CD: Celiac Disease; GI: Gastrointestinal

**Table 6.** Our results comparison with low resolution commercially available kits: Sample number 3 and 16 was false negative and sensitivity/specificity was 80 and 100 percentage, respectively

		HLA typing with commercially available kits	
		HLA typing with Olerup kit	HLA typing with Morgan kit
Number of patients		20	20
DQ2	sensitivity	80	80
	specificity	100	100
DQ8	sensitivity	80	80
	specificity	100	100

**Validation of real-time PCR for detecting DQ2 and DQ8**

To verify result from Real-time PCR, we re-evaluate the HLA alleles for 20 CD cases and 20 controls by using two different available commercial kits (Olerup SSP HLA typing kit, Saltsjöbaden, Sweden. and MorganTM HLA SSP A, B, C, DR, DQ Typing Kit, Texas, BioGene, Inc, USA.) which are commonly used in general diagnostic laboratories in Iran. Accordingly, HLA-DQ2, HLA-DQ8 and HLA-DQ2.5 was determined and compared to our results.

**Statistical analysis**

The frequency of HLA alleles was performed using SPSS v.16 (SPSS Inc, Chicago, IL). Sensitivity and specificity were determined using the following formula (Sensitivity = (Number of true positive) / (Number of true positive+ Number of false positive), Specificity =(Number of true negative )/(Number of true negative+ Number of false positive) ). A P-value less than 0.05 was considered statistically significant. 

## Results

**Demographic data**

Seventy-five CD patients and 94 controls were enrolled in this study. Different GI and extra GI symptoms were reported in the case group. The most prevalent GI symptoms in these patients were heartburn (69.4%), diarrhea (48%), nausea and vomiting (40%) and also the most extra-GI symptom included weight loss (68%), anemia (57.4%) and aphthous (49.4%). As well as, in celiac group 32 patients had family history of CD. The histology data revealed 7 patients with Marsh-I, 9 patients with Marsh-II, 59 patients with Marsh-III. Details of demographic data are briefly shown in Table 5.

**Conventional PCR and Temperature gradient**

**Figure 1:**
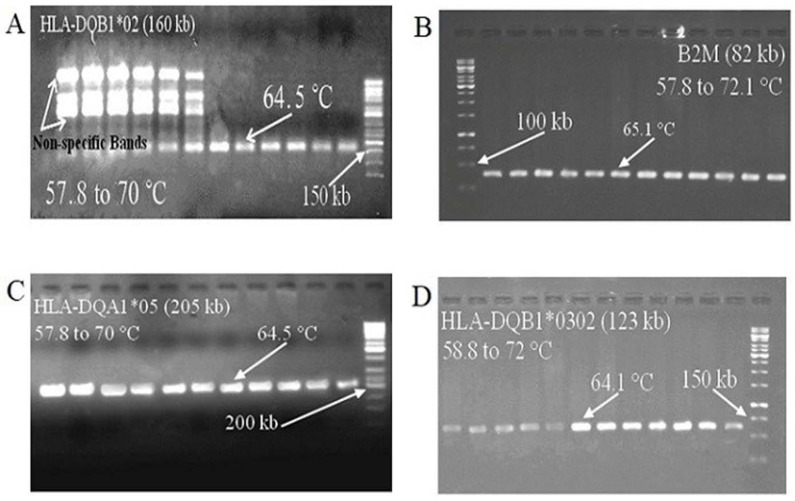
The results of the temperature gradient for each primer; the reaction temperature range and length of each primer fragment have been shown. All primers had amplified in temperatures 65 °C. A: HLA-DQB1*02, in low temperatures this primer amplified a non-specific band, but these bands were removed with increasing temperature; B: B2M; C: HLA-DQA1*05; encoding the Alpha chain in DQ2.5 sub-allele; D: HLA-DQB1*0302, DQ8 encoding

Temperature gradient was performed for each primer pairs including HLA-DQA1*02, HLA-DQB1*0302, HLA-DQA1*05 and B2M individually and the length of each amplicon was 160 bp (base pair), 123 bp, 205 bp and 82 bp, respectively. Accordingly, the correct Tm for each primer was set around 65^ °^C. The results were shown in Figure 1. 

**Melting curve analysis data**

**Figure 2:**
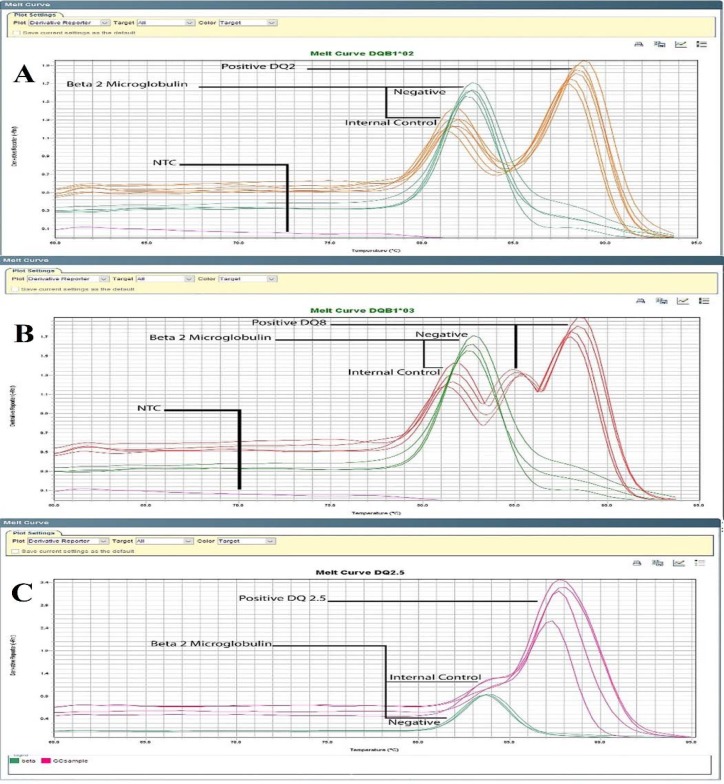
Specific melting curve of each allele; Average of Melting temperature was 81.8 °C (B2M), 84.5 (HLA-DQA1*05), 88.1 °C (HLA-DQA1*02), 89.2 °C (HLA-DQB1*0302). A: DQ2 positive samples have two peaks of HLA-DQB1*02 and B2M but DQ2 negative cases only have peak of Internal control; B: Such as status can be observed for DQ8 positive sample that carried HLA-DQB1*0302 allele; C: Among DQ2 positive cases who those carried DQ2.5 allele categorized in high risk group. The peak of internal control (B2M) and HLA-DQA1*05 allele are in close range, but considering the overall Tm differences between them, DQA1*05 positive samples can be distinguished from negative samples

Based on foundation of Melting curve analysis in Real-time PCR SYBR Green, two peaks could be observed on curve diagram, that first peak belongs to internal control and second peak was related to alleles (Figure 2). HLA-DQ 2.5 allele had close Tm peak to internal control, (Figure 2C). Average of Melting temperature for B2M was 81.8 ^°^C, HLA-DQA1*05: 84.5 ^°^C, HLA-DQA1*02: 88.1 ^°^C, and HLA-DQB1*0302: 89.2 ^°^C.

**Sensitivity and Specificity**

Twenty cases and 20 controls were randomly selected and re-genotyped by commercial kits. The sensitivity and specificity of this method compared to low resolution commercially available kits was 80 and 100 percentages, respectively. Sample number 3 and 16 were false negative for DQ8 but all samples matched DQ2 (Table 6).

**Allele frequency of HLA-DQ2 and HLA-DQ8 in cases and controls**

According to the result of this new method 76% and 29% of the CD patients and 53.2% and 44.7% of control carried HLA-DQ2 and DQ8 haplotypes respectively. Overall 96% of patients carries DQ2 and/or DQ8 and compared to control (52.1 %), this difference was statistically significant (P = 0.001). 

## Discussion

Genetic predispositions, immunological and environmental are key factors in CD. The environmental factor for development of CD is dietary exposure to gluten peptides in wheat, rye and barley and presence of HLA DQ2/8 haplotypes. Genetic studies showed the role of HLA class II (HLA-DQ) as necessary but insufficient determinant of developing CD (9, 11, 12). Approximately 90% of CD patients are DQ2 positive and most of the remaining 10% have a DQ8 heterodimers (2).

HLA-DQ2/8 typing helps to rule out CD in suspected patients like first degree relatives and those patients who are at low risk of having CD, when serology or biopsy are inconclusive (14, 17). Therefore, HLA typing has become an essential additional screening tool for diagnosis of CD in atypical patients (18).

The HLA typing as diagnostic tool for CD, has been developed using different methodologies. Most of these methods are out of date. These diagnostic methods have advantages and disadvantages. For example, two decades ago, Otten and colleagues used the serological method for HLA-DQ typing. This method has different difficulties and also low accuracy (19). With the rise of molecular approaches, Michalski et al. used Restriction Fragment Length Polymorphism-PCR (RFLP-PCR) technique to determine HLA-DQ haplotypes in CD patients (20). Although this method has high accuracy compared to serological method, but it was time-consuming, laborious and needed sequencing for verification. After that, Olerup et al. designed Single Specific Primer-PCR (SSP-PCR) technique which was able to detect all HLA-DQ alleles (21). SSP-PCR method is sensitive approach but it has required post-PCR process. Later, Megiorni et al. determined HLA-DQ alleles in patients with CD using Olerup´s designed primers (13). As these primers have overlap with other haplotypes such as DQ3, DQ7 and DQ9, there was possibility of false positive results. 

Monsuur et al. used TaqMan Probe to determine HLA-DQ2/8 alleles in CD patients (22). TaqMan Probe has acceptable precision but it is very expensive. Another molecular technique that recently used for HLA typing are including Sequence-Based Typing (SBT) method (23), Sequence-Specific Oligonucleotide Probe Hybridization (SSOPH) (24), Reference Strand-mediated Conformation Analysis (RSCA) (25) and PCR-SSP (26). These methods provide powerful tools to determine HLA alleles but they have limitations such as high costs (RSCA), laborious (SSOPH), and unsuitable for large-scale (SBT) population. Many of these methods were optimized and came into the market as a commercial kit such as Olerup SSP-Kits, but they are expensive.

In this study, in order to simplify determination of HLA-DQ2/8 alleles we used Real-time PCR SYBR Green with melting curve analysis as used previously by T. Profaizer et al. (27). They used Human Growth Factor’s primer as an internal control but as it had same Tm with HLA-DQ alleles and test was not repeatable (27), so we used optimal primers for B2M that were repeatable and had different Tm with HLA-DQ alleles. 

**Figure 3:**
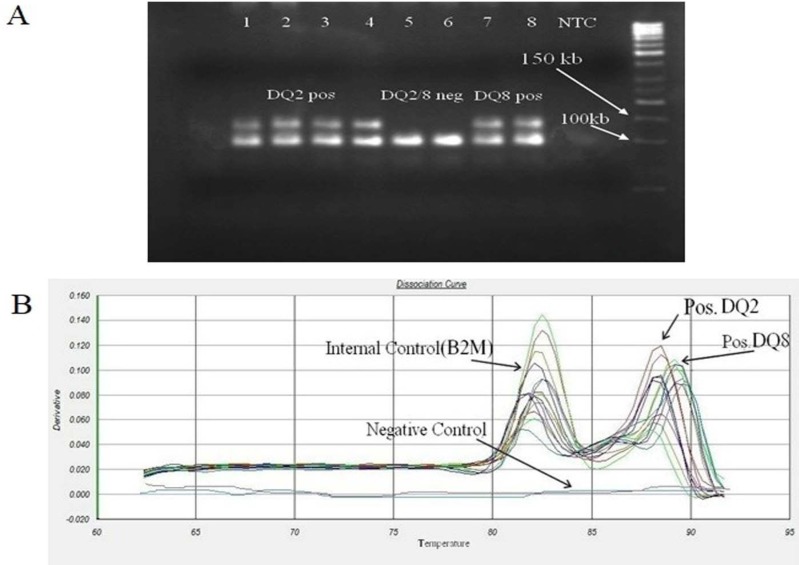
Similarity of specific and non-specific HLA-DQB1*0302 primer amplicon for DQ2 and DQ8 positive samples. A: DQB1*0302 primers amplified DQ2 positive samples and produced non-specific amplicon that this non-specific band similar to DQ8 products and they are not distinguished on gel electrophoresis. Samples 1-4 were DQ2 positive and produce non-specific amplicon by DQB1*0302 primers that they are not distinguished from DQ8 positive sample (samples 7 and 8) on gel electrophoresis. Samples 5 and 6 were negative control; B: Specific and non-specific amplicon were separable by melting curve analysis due to differences between melting temperatures of each products

Compared to the conventional methods, our method is simple, faster, cost effective and has high sensitivity and specificity. From a practical standpoint, mixed reaction can be pre-aliquoted and stored in the freezer (As a commercial kit) and only patient's DNA need to be added and this increases the easiness of the assay. With the dismissal of the need to view post-PCR products by gel electrophoresis, the amount of sample handlings is reduced, so this technique will reduce the risks of sample mix-up. Furthermore, it eliminates exposure to toxic dyes that will make this method a desirable clinical test for the diagnosis of CD for large scale studies.

Our results show that melting curve peaks of internal control (B2M) and HLA-DQA1*05 allele are in close range, but considering the overall Tm differences between them, DQA1*05 positive samples can be distinguished from negative samples (Figure 2C). In other hand, we noticed that the DQ8 primers amplified DQ2 positive samples and therefore produced non-specific amplicon. This non-specific band is similar to DQ8 products and not distinguishable on gel electrophoresis (Figure 3A) but due to differences between melting temperatures of each products, the fragments were separable by melting curve analysis. In order to facilitate recognition of these products from each other, we used DQ2 and DQ8 positive samples (as a positive control) in each round and then results of other samples were compared to them (Figure 3B). The main disadvantage of this method is that it requires expensive equipment that are not available in all laboratories.

Few studies were reported the frequency of DQ2/8 alleles in Iranian normal population and revealed that alleles frequency of DQ2 and/or DQ8 were 40-60 percentages (28-32). The first study on the prevalence and association of HLA-DQ2 and HLA-DQ8 haplotypes with CD in Iran by Rostami-Nejad et al. indicated that 97% of patients were carriers of HLA-DQ2 and/or -DQ8 haplotypes (6). Our study with this new method also confirmed that around 96% of CD were carriers of DQ2 and/or DQ8 haplotypes. Also similar to Rostami-Nejad and colleagues findings, our results revealed that most of DQ2 positive patients (61.3%) carry DQ2.5 allele indicating a high risk for CD compared to other patients (Results are not shown) (6).

Finally, Real-time PCR using SYBR Green method with melting curve analysis has a better efficiency, is faster, more cost effective, easier to use and has higher sensitivity and specificity to distinguish these alleles compared to conventional HLA-typing techniques in identifying the HLA-DQ2 and HLA-DQ8 alleles in patients with atypical CD or those at high risk including the first-degree relatives of CD patients.
